# The influence of peer support on college students’ physical exercise: the mediator of control beliefs and subjective experience, and the moderator of intrinsic value

**DOI:** 10.3389/fpsyg.2026.1711318

**Published:** 2026-02-12

**Authors:** Feng-Qing Jiang, Ke Ni, Ting Zhang, Jian-Ping Wang

**Affiliations:** 1 College of Sports and Art, Jiangxi University of Science and Technology, Ganzhou, China; 2 Physical Education College, Jiangxi Normal University, Nanchang, China

**Keywords:** control beliefs, intrinsic value, peer support, physical exercise, subjective exercise experience

## Abstract

College students’ physical exercise levels remain suboptimal despite its well-documented health benefits. Peer support, as a key form of social influence, has been shown to facilitate health behaviors, yet the underlying psychological mechanisms—particularly the roles of control beliefs and subjective exercise experience—remain underexplored. To address this gap, this study involved 1,510 college students to investigate how peer support influences physical exercise through these psychological pathways, with intrinsic value examined as a potential moderator. The research employed multiple measurement tools, including scales assessing peer support, control beliefs, subjective exercise experience, intrinsic value, and physical exercise. Key outcomes showed that: (1) Peer support is directly associated with increased physical exercise, while also working through three indirect pathways: via its positive associations with students’ sense of control beliefs and subjective exercise experience, and through a combined effect of both control beliefs and subjective exercise experience. (2) Intrinsic value moderates (i.e., strengthens) the association between control beliefs and subjective exercise experience, as well as the relationships of both control beliefs and subjective exercise experience with physical exercise. These insights advance our understanding of social influences on student fitness behaviors and provide actionable suggestions for universities aiming to increase student engagement in physical activities.

## Introduction

Physical exercise serves as an extension of school-based physical education, encompassing diverse forms of movement that students engage in during their free time. These activities, primarily focused on health maintenance, leisure enjoyment, and recreational purposes, can be measured through parameters such as participation frequency, exercise intensity, and duration (Cheng and Dong, 2018). Research indicates that students frequently participate in sports activities, which can enhance physical fitness, reduce obesity risks, and boost cardiovascular endurance (Zhang et al., 2025). However, due to the comprehensive influence of the external environment, cognitive development level, interpersonal perception ability, etc., college students’ physical exercise is still characterized by small intensity, low frequency, and short duration (Dong and Mao, 2018).

Peer support, as a form of social support, plays a crucial role in facilitating individuals’ socialization and fostering the adoption of healthy behaviors (Efrat, 2009; Menescardi and Estevan, 2021). At the college level, students spend most of their time in college, and the influence of peers on individuals gradually increases, so peer support becomes the most important source of socialization support and is important for them to improve their physical exercise (Chen, 2020). Academic research has predominantly focused on college student populations when examining how peer support influences participation in physical exercise (Liu, 2024; Chen, 2022). Nevertheless, existing literature has not systematically examined the underlying mechanisms by which peer support affects physical exercise engagement among college students, nor has it comprehensively investigated potential moderators of these relationships. To address this gap, the current study aims to develop and test a moderated mediation model that: (1) identifies multiple mediating pathways linking peer support with physical exercise, and (2) assesses how individual characteristics may differentially moderate these pathways. The findings are expected to offer both theoretical insights and practical implications for enhancing physical exercise participation in higher education settings.

### The association between peer support and physical exercise

College students’ engagement in physical exercise is significantly shaped by their social support networks (Gruber, 2008), with peer groups emerging as the primary source of such support for exercise behaviors (Zou et al., 2023). Peer support refers to the multidimensional encouragement that close social networks provide students, encompassing cognitive motivation, emotional reinforcement, and behavioral engagement in physical exercise (Liu, 2024). Yang (2017) showed that “friends’ encouragement and participation” and “friends’ invitation and praise” were positively correlated with college students’ performance of moderate-to-vigorous intensity exercise. Another study by Zhang et al. (2022) found that college students with higher peer support scores had better physical exercise. Zhang et al. (2022) revealed a positive association between perceived peer support levels and physical exercise engagement among college students. These findings suggest peer support may be linked to physical exercise engagement among college students through multiple mechanisms including companionship, positive reinforcement, motivational encouragement, and behavioral modeling. Accordingly, we hypothesize (H1) that peer support positively is associated with physical exercise in college students.

### The mediating effect of control beliefs

Control-value theory (Pekrun, 2006) conceptualizes control beliefs as cognitive appraisals of one’s capacity to execute behaviors and affect desired outcomes, operationalized through self-regulatory constructs including self-efficacy, self-concept, and self-control. Combing through the existing literature, it was found that few studies directly focused on the theme of peer support and control beliefs, but peer support is inextricably linked to self-control, self-efficacy, and self-concept (Liu, 2024), which indirectly reflects a strong correlation to control beliefs. For example, Chen (2022) found that peer support helped improve self-control among rural left-behind students. Chen et al. (2017) also found that peer support was highly predictive of students’ self-efficacy. Perceived peer support was positively predictive of adolescents’ general self-concept (Fernández-Zabala et al., 2020). Empirical studies have identified control beliefs as a key determinant of sustained physical exercise participation (Kaushal et al., 2021). Established empirical studies have demonstrated a positive association between control belief and exercise engagement, including participation initiative, motivational levels, and regimen adherence (Dong and Mao, 2018). Wang et al. (2018) found that support and advice from peers can increase an individual’s self-efficacy, which increases their physical exercise. Prior studies revealed that peer-provided emotional support significantly enhanced students’ exercise self-efficacy and demonstrated particularly strong associations with physical exercise (Wang et al., 2023). Building on these findings, we hypothesize (H2) that control beliefs mediate the association between peer support and physical exercise among college students.

### The mediating effect of subjective exercise experience

Researchers have found that subjective exercise experience, which acts as an irrational factor, is correlated with physical exercise when attributing adolescent exercise behavior (Kwan and Bryan, 2010). This concept describes a person’s assessment of their own past emotional experiences related to physical exercise, whether positive or negative (Zhang et al., 2023). Positive exercise experiences may be assimilated as inherent drive, cultivating exercise intentions motivated by hedonic pursuits (He et al., 2022). However, individuals who lack positive experiences or have negative exercise experiences, and have sustained negative cognitive responses to exercise behaviors tend to exhibit rejection and resistance tendencies that severely limit physical exercise (Chen et al., 2006). Previous research has confirmed that positive subjective exercise experience is significantly correlated with both exercise engagement and adherence behaviors (Tian and Shi, 2022). Social factors, particularly interpersonal support networks, have been shown to shape exercise-related affect. Empirical evidence suggests that both instrumental and emotional support from significant others can mitigate negative exercise perceptions while fostering positive affective responses (Siedlecki et al., 2014). Studies have found that the association between social support and exercise maintenance is mediated by individuals’ subjective exercise experience (Tian and Shi, 2022). Peer support serves as a crucial social determinant that enhances college students’ sense of pleasure during physical exercise, resulting in more positive emotional experiences (Wang et al., 2023). Existing studies have found that peer support positively predicts adolescents’ exercise enjoyment (Chen et al., 2017). Building on these findings, we hypothesize (H3) that subjective exercise experience may mediate the association between peer support and physical exercise.

### The chain mediating role of controlling beliefs and subjective exercise experience

Control beliefs and subjective exercise experience explain a person’s perceived competence and subjective emotional experience during exercise, respectively (Dong and Mao, 2018; Tian and Shi, 2022). Dual-process theory suggests that both perceived competence and emotional responses are required to engage/inhibit desired behaviors (Jang et al., 2016). Control beliefs are a type of control evaluation. According to control-value theory, the perceived level of control appraisal of a social behavior effectively predicts an individual’s emotional experience of behavior (Pekrun and Perry, 2014). It is also said that when individuals perceive that a behavior has a high control appraisal, they show a more positive emotional experience and better participate in the behavior (Zhang and Li, 2023). Simonton et al. (2022) found that control beliefs indirectly influenced physical activity participation via enjoyment mediation. Meanwhile, this theory suggests that the external environment is influencing emotions by affecting control appraisals and ultimately having an impact on behavior. Zhang and Li (2023) found that teacher autonomy support influences enjoyment through academic control and ultimately influences physical exercise in adolescents. Although peer support has not been involved in the sequential mediation of control beliefs and subjective exercise experience in influencing physical exercise, research has indicated that it exerts an indirect effect on adolescent exercise participation through the sequential mediation of enjoyment and self-efficacy (Chen et al., 2017). Building on these findings, we hypothesize (H4) that control beliefs and subjective exercise experience may sequentially mediate the association between peer support and physical exercise.

### The moderating role of intrinsic value

Intrinsic value reflects the inherent enjoyment an individual derives from task engagement, showing a particularly active intrinsic motivation, so as to enjoy the pleasure and value brought by this task (Eccles and Wigfield, 2002). Ternary interaction theory suggests that there is an interaction between environment, cognition and behavior (Bandura, 2001), which provides a theoretical basis for the correlation of intrinsic value with peer support and physical exercise. Empirical evidence has indicated that intrinsic motivation is positively associated with both peer support (Tilga et al., 2018) and exercise (Staples et al., 2022). According to control-value theory, control beliefs and value beliefs are not only closely related as motivational beliefs (Garn and Simonton, 2022), but also jointly influence emotions (Garn and Simonton, 2022), which provides a theoretical basis for the association of intrinsic value with both control beliefs and subjective exercise experience. Empirical evidence has demonstrated that intrinsic value is positively associated with control beliefs (Simonton and Garn, 2020) and subjective exercise experience (Liu and Li, 2023). However, the potential moderating role of intrinsic value in the mechanistic pathways linking peer support, control beliefs, subjective exercise experience, and physical exercise requires empirical verification. Building on these findings, we propose the following moderated relationships (H5): H5a. The relationship between peer support on control beliefs, subjective exercise experience, and physical exercise is moderated by intrinsic value, respectively; H5b. The relationship between control beliefs on subjective exercise experience and physical exercise is moderated by intrinsic value, respectively; H5c. The relationship between subjective exercise experience on physical exercise is moderated by intrinsic value.

## Methods

### Participants

The study involved five regular universities in China. Among students in grades 1–3, a convenience sampling method was used to select 15 classes in each grade. These universities were located in northern, eastern, and southern China, and included both comprehensive and technology-focused institutions, aiming to diversify the sample in terms of region and university type. A total of 1,705 students from these classes voluntarily participated in the anonymous survey. Invalid responses were identified and excluded based on the following criteria: (1) blank or incomplete questionnaires; (2) response regularity patterns (e.g., repeated sequences such as 1, 2, 3, 4, 5, 1, 2, 3, 4, 5; identical scores across all items; or straight-line responding). After removing invalid responses, 1,510 valid questionnaires were retained from the initial 1,705 (*M*_age_ = 19.14, SD_age_ = 1.06), yielding an effective rate of 88.56%. The sample consisted of 48.1% male and 51.9% female participants. By academic year, grade 1, grade 2, and grade 3 accounted for 38.5, 27.5, and 33.8%, respectively. Both the college students and their parents gave written informed consent prior to participation.

### Measures

#### Intrinsic value scale

The intrinsic value scale, originally developed by Schiefele et al. (1988), assesses personal interest through three unidimensional items. Zhang and Li (2023) adapted it into Chinese and confirmed its reliability and validity. The term “physical activity” was modified to “physical exercise” (e.g., “I enjoy engaging in physical exercise during my free time”). Responses were recorded on a 5-point Likert scale (1 = strongly disagree, 5 = strongly agree). The scale demonstrated high internal consistency (Cronbach’s α = 0.914). Total scores were calculated to reflect intrinsic value levels, with higher scores indicating greater intrinsic motivation. Previous research (Zhang and Li, 2023) supports its reliability and cultural suitability for Chinese populations.

#### Control beliefs scale

The physical activity self-concept subscale, originally developed by Marsh et al. (2010) as part of the Physical Self-Description Questionnaire, was adapted into Chinese by Zhang and Li (2023). This adapted version has demonstrated satisfactory psychometric properties, including reliability and validity. The subscale measures individuals’ perceived competence in maintaining consistent physical exercise routines (Garn and Simonton, 2022). In the Chinese version, the term “physical activity” was modified to “physical exercise” to better suit the cultural context, as seen in items like “I engage in physical exercise (such as jogging, dancing, cycling, aerobics, gym workouts, or swimming) at least three times weekly.” Comprising four items, the subscale employs a 5-point Likert response format, ranging from 1 (“strongly disagree”) to 5 (“strongly agree”). The scale’s structural validity was supported by confirmatory factor analysis, with factor loadings between 0.670 and 0.844. Fit indices (χ^2^/df = 5.268, NFI = 0.997, GFI = 0.998, CFI = 0.998, RMSEA = 0.057) demonstrated reasonable model fit. Reliability analysis yielded a Cronbach’s α of 0.842, indicating strong consistency. Exercise-related self-efficacy was quantified by summing item scores, where higher totals indicated stronger self-efficacy.

#### Subjective exercise experience scale

The study employed the revised subjective exercise experience scale (Cheng and Dong, 2018), which includes two subscales: positive well-being (4 items) and psychological distress (4 items). Responses were recorded on a 5-point Likert scale (1 = “very non-compliant” to 5 = “very compliant”). The measurement model was validated through confirmatory factor analysis, revealing robust factor loadings ranging from 0.808 to 0.924. Model fit statistics (χ^2^/df = 3.841, NFI = 0.994, GFI = 0.991, CFI = 0.995, RMSEA = 0.047) indicated satisfactory fit to the data. The internal consistency reliability of each scale was excellent (α = 0.922 and 0.918). Composite scores, derived from item summation, reflected the intensity of students’ positive exercise perceptions.

#### Peer support scale

Peer support was measured using a validated 5-item subscale (Yang, 2016), featuring items such as “My peers encourage my physical activity participation.” Responses were collected using a 5-point agreement scale. Validation analyses confirmed strong psychometric properties: all items loaded significantly (0.668–0.884), model fit indices were excellent (χ^2^/df = 2.046, NFI = 0.997, GFI = 0.997, CFI = 0.999, RMSEA = 0.028), and reliability was high (α = 0.875). The total score, calculated by summing item responses, served as an indicator of peer support intensity.

#### Physical exercise rating scale

The study employed Liang’s (1994) validated physical exercise assessment tool, which examines three key exercise components: intensity, frequency, and duration. Using 5-point rating scales for each dimension, an overall exercise score (0–100 range) was calculated by multiplying intensity by adjusted duration (time - 1) and frequency. This measure has demonstrated strong reliability in prior research (*r* = 0.82).

#### Data analysis

Before conducting the analysis, we performed normality tests on all continuous variables (with the absolute values of skewness and kurtosis both less than 2), and checked for homogeneity of variance (the residual plot showed no significant heteroscedasticity). The multicollinearity diagnosis revealed that the variance inflation factor (VIF) of all predictor variables was less than 5, indicating no serious collinearity issues. To examine the hypothesized mediation and moderation effects, Hayes (2013) PROCESS macro (v3.5) was employed, specifically utilizing Model 6 for chain mediation and Model 92 for moderated effects (significance threshold: *p* < 0.05). The bias-corrected bootstrap method (5,000 resamples, 95% CIs) was applied, with mediation effects deemed significant if the confidence intervals excluded zero.

## Results

### Common method bias assessment

To evaluate potential common method variance resulting from self-report measures, we employed Harman’s single-factor test. Principal component analysis without rotation extracted five components with eigenvalues >1, where the primary component explained 39.04% of total variance - falling below the critical 40% cutoff. Subsequently, Amos 26.0 was used to perform a single-factor confirmatory factor analysis, with all items loaded onto a single-factor model. The model fit indices were as follows: χ^2^/df = 46.108, NFI = 0.574, GFI = 0.529, CFI = 0.579, RMSEA = 0.173, indicating poor model fit. Overall, common method bias in this study is not significant.

### Summary statistics and bivariate correlations

The correlation analysis presented in Table 1 demonstrates several significant relationships among key variables. Peer support showed positive correlations with control beliefs, subjective exercise experience, intrinsic value, and physical exercise. Similarly, control beliefs were positively associated with subjective exercise experience, intrinsic value, and physical exercise. Additional positive correlations emerged between subjective exercise experience and both intrinsic value and physical exercise, as well as between intrinsic value and physical exercise. Regarding demographic factors, peer support correlated positively with household income and physical fitness test, while control beliefs exhibited negative associations with gender and registered residence but positive relationships with academic grade and physical fitness test. Subjective exercise experience was inversely related to gender but positively linked to household income and physical fitness test. Both intrinsic value and physical exercise showed negative correlations with gender, while maintaining positive associations with physical fitness test. Physical exercise additionally demonstrated a positive relationship with household income.

**Table 1:** Bivariate correlation statistics.

Dimension	*M* ± SD	Gender	Grade	RR	IH	PF	PS	CB	SEE	IV	PE
Gender	0.52 ± 0.50	1									
Grade	1.96 ± 0.85	−0.051*	1								
RR	0.43 ± 0.50	0.012	−0.069**	1							
IH	3.88 ± 1.58	−0.048	−0.001	0.250**	1						
PF	3.00 ± 1.02	0.130**	−0.080**	−0.043	0.059*	1					
PS	16.34 ± 4.53	0.000	−0.012	−0.017	0.064*	0.214**	1				
CB	12.62 ± 3.77	−0.120**	0.138**	−0.079**	0.036	0.235**	0.525**	1			
SEE	29.77 ± 5.70	−0.121**	−0.032	0.036	0.077**	0.240**	0.407**	0.458**	1		
IV	9.56 ± 2.91	−0.154**	−0.039	−0.022	0.044	0.306**	0.531**	0.653**	0.616**	1	
PE	18.00 ± 16.08	−0.186**	0.001	−0.006	0.119**	0.220**	0.288**	0.398**	0.363**	0.448**	1

*p < 0.05, **p < 0.01; RR, Registered residence; IH, Income of households; PF, Physical fitness test; PS, peer support; CB, control beliefs; SEE, subjective exercise experience; IV, intrinsic value; PE, physical exercise (the same below).

### Moderated mediation model testing

Following Wen and Ye’s (2014) recommendations, all variables were standardized prior to analysis. The moderated mediation analysis was conducted in two stages, controlling for demographic covariates (gender, grade, residence, household income, and physical fitness) (Table 2).

**Table 2:** Multiple mediation regression modeling.

Dimension	PE			CB			SEE			PE		
	β	SE	*t*	β	SE	*t*	β	SE	*t*	β	SE	*t*
Gender	−0.206	0.024	−8.633^***^	−0.132	0.021	−6.214^***^	−0.102	0.022	−4.554^***^	−0.150	0.023	−6.444^***^
Grade	0.008	0.024	0.320	0.146	0.021	6.912^***^	−0.063	0.022	−2.820^**^	−0.025	0.023	−1.065
RR	−0.012	0.025	−0.469	−0.052	0.022	−2.381^*^	0.061	0.023	2.697^**^	−0.007	0.024	−0.293
IH	0.086	0.025	3.491^**^	0.002	0.022	0.092	0.025	0.023	1.088	0.081	0.023	3.456^**^
PF	0.191	0.025	7.758^***^	0.157	0.022	7.161^***^	0.130	0.023	5.604^***^	0.122	0.024	5.067^***^
PS	0.242	0.024	9.973^***^	0.492	0.022	22.814^***^	0.212	0.026	8.152^***^	0.059	0.027	2.172^*^
CB							0.316	0.027	11.790^***^	0.240	0.029	8.320^***^
SEE										0.175	0.027	6.582^***^
*R^2^*	0.160			0.335			0.281			0.240		
*F*	47.87^***^			126.38^***^			83.838^***^			59.345^***^		

*p < 0.05, **p < 0.01, ***p < 0.001 (the same below).

In the first step, the hypothesized chain mediation model was tested using Hayes’ PROCESS Model 6 in SPSS 20.0, controlling for relevant covariates. Initial analysis revealed peer support showed a significant total association with physical exercise (β = 0.242, *p* < 0.001). After introducing mediators, the direct effect remained statistically significant (β = 0.059, *p* < 0.05), confirming Hypothesis 1. Path analysis showed peer support was significantly associated with both proposed mediators: control beliefs (β = 0.492, *p* < 0.001) and subjective exercise experience (β = 0.212, *p* < 0.001). Notably, control beliefs were positively associated with subjective exercise experience (β = 0.316, *p* < 0.001), and both mediators showed significant associations with the outcome variable (control beliefs: β = 0.240; subjective exercise experience: β = 0.175; both *p* < 0.001). These results substantiate the proposed sequential mediation pathway.

The mediation analysis results (see Table 3) revealed three significant pathways: (1) the control beliefs mediation path (effect = 0.118, BootSE = 0.016, 95%CI [0.087, 0.150]); (2) the subjective exercise experience mediation path (effect = 0.037, BootSE = 0.008, 95%CI [0.023, 0.053]); and (3) the sequential mediation path through both mediators (effect = 0.027, BootSE = 0.005, 95%CI [0.018, 0.038]). These findings confirm that peer support influences physical exercise through three distinct mechanisms: independently via control beliefs, independently via subjective exercise experience, and sequentially through both mediators, thereby supporting Hypotheses 2–4. Notably, the direct effect remained significant (effect = 0.059, BootSE = 0.028, 95%CI [0.006, 0.115]), indicating partial mediation by the proposed psychological mechanisms.

**Table 3:** Examination of CB and SEE as mediators.

Dimension	Effect size	BootSE	Boot95%CI	
			Lower	Upper
Total effect	0.241	0.024	0.194	0.289
Direct effect	0.059	0.028	0.006	0.115
Total mediating effect	0.182	0.018	0.148	0.218
PS → CB → PE (Med1)	0.118	0.016	0.087	0.150
PS → SEE → PE (Med2)	0.037	0.008	0.023	0.053
PS → CB → SEE → PE (Med3)	0.027	0.005	0.018	0.038

**Figure 1:**
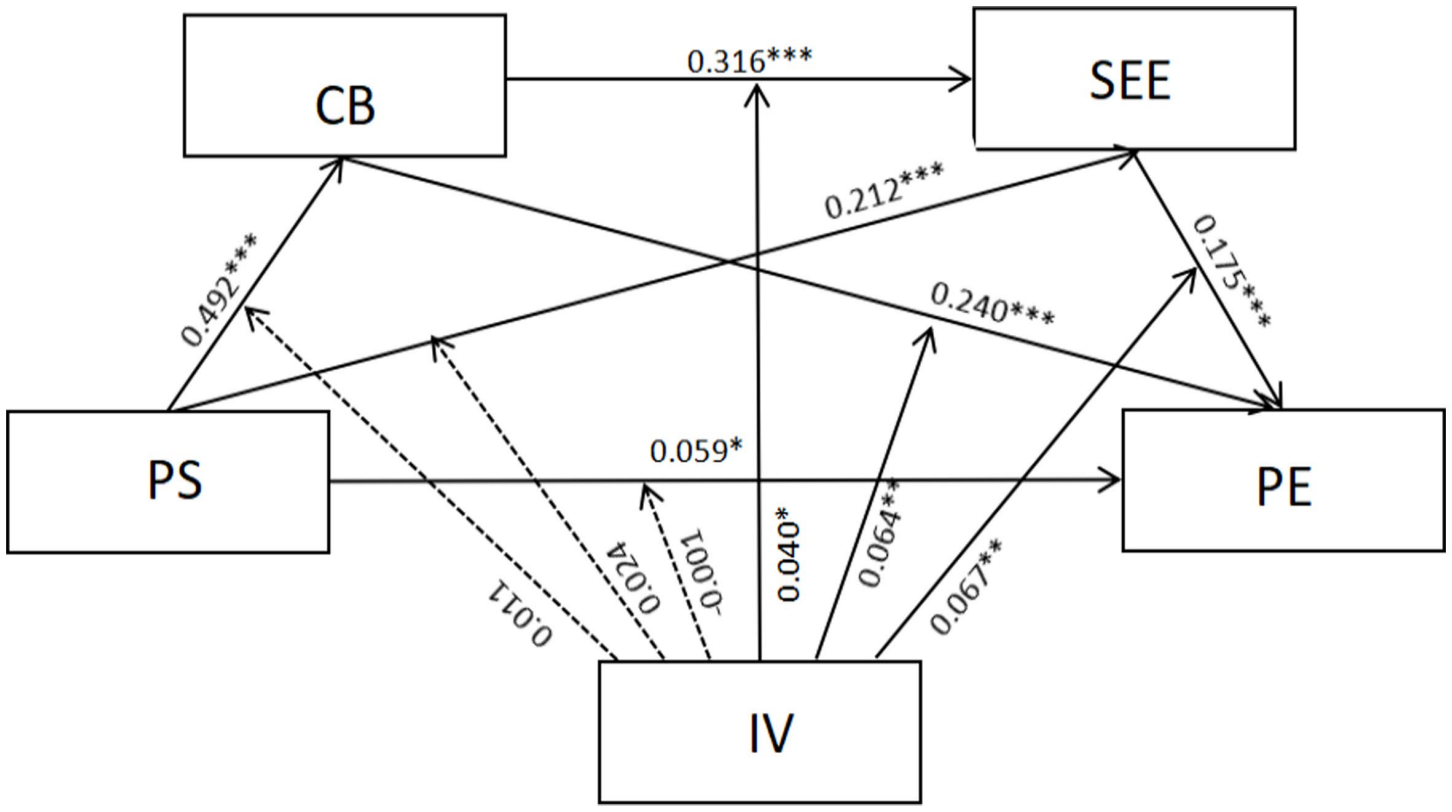
The model of moderated chain mediating effect.

**Figure 2:**
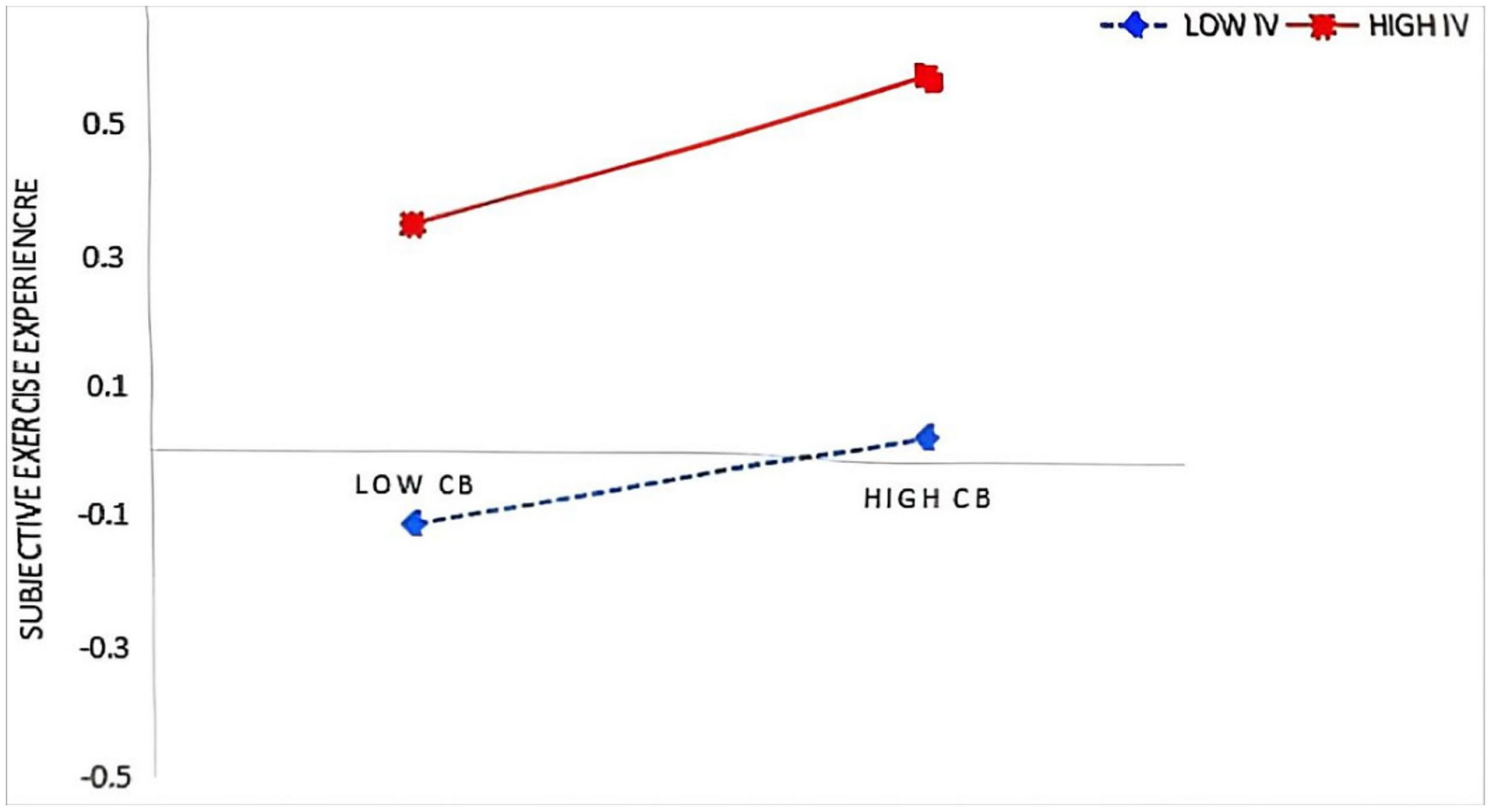
CB × IV interaction on SEE.

**Figure 3:**
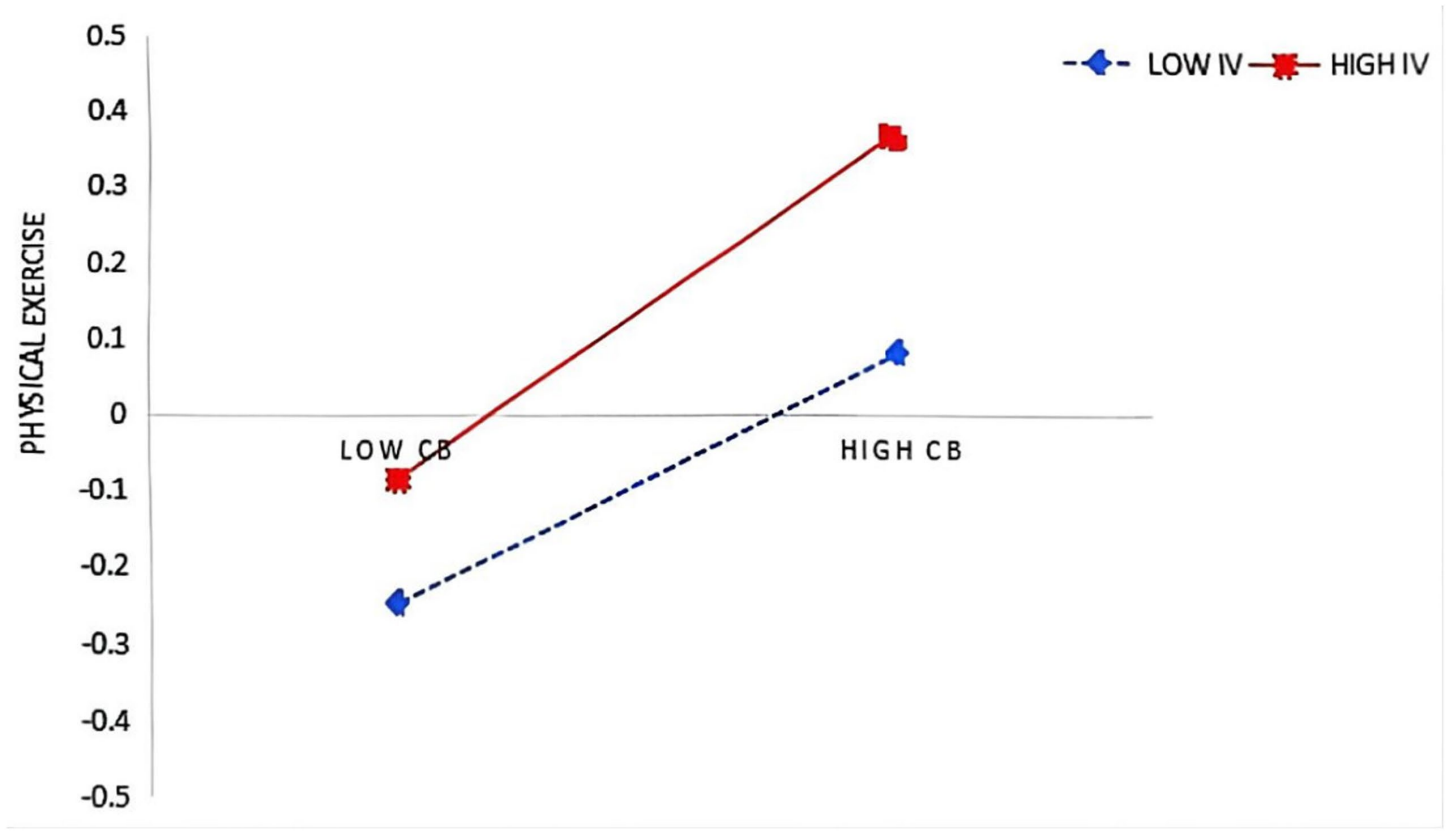
CB × IV interaction on PS.

**Figure 4:**
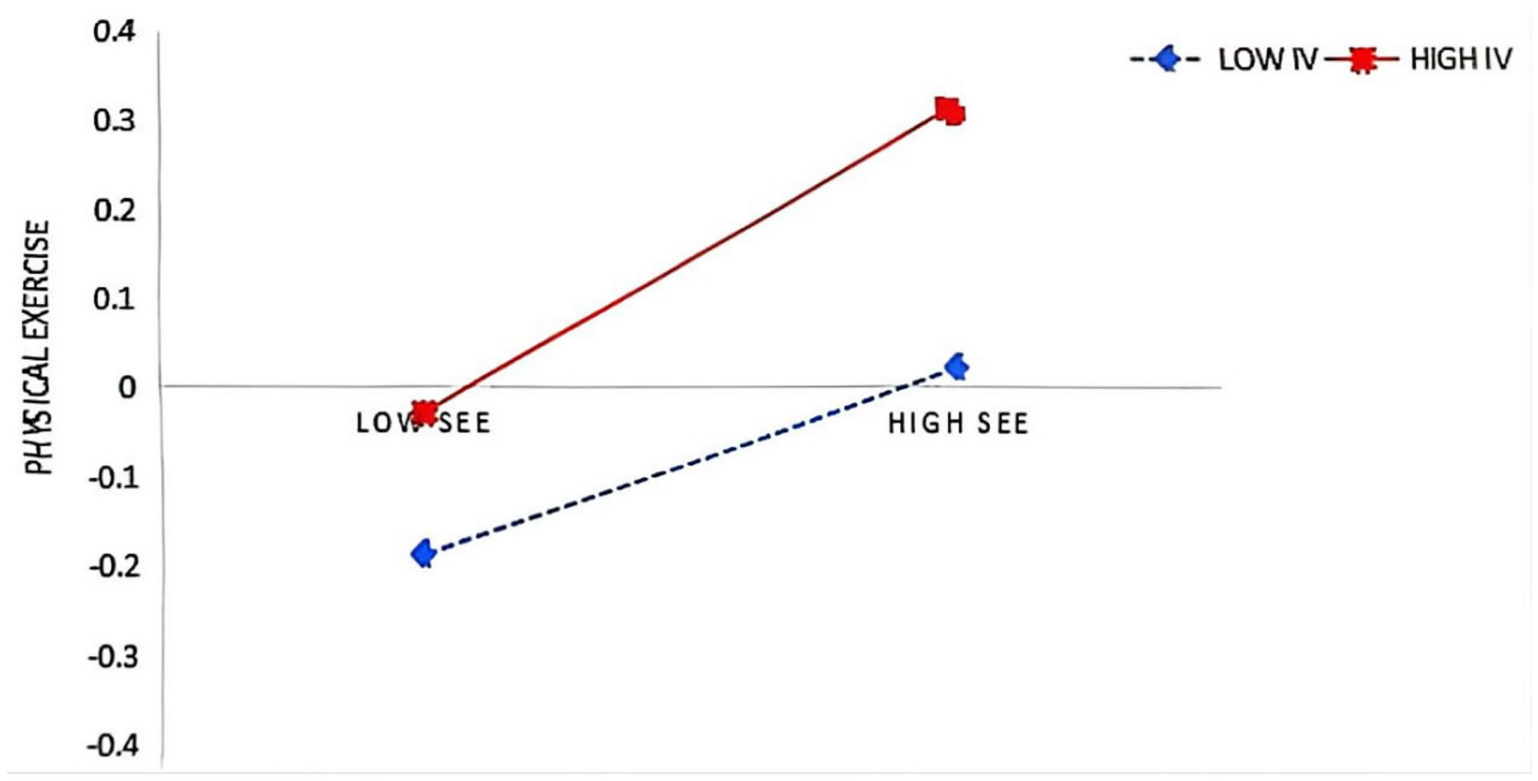
SEE × IV interaction on PS.

In the second step, the moderation analysis using PROCESS Model 92 revealed that intrinsic value significantly enhanced (see Table 4; Figure 1): (1) the effect of control beliefs on subjective exercise experience (β = 0.040, *p* < 0.05), and (2) the effects of both control beliefs (β = 0.064, *p* < 0.01) and subjective exercise experience (β = 0.067, *p* < 0.01) on physical exercise, supporting Hypotheses 5, 5b, and 5c. However, intrinsic value did not moderate the relationships between peer support and other variables (*p* > 0.05), rejecting Hypothesis 5a. Simple slope tests were conducted by categorizing intrinsic value into high (M + 1SD) and low (M − 1SD) groups based on ±1 standard deviation from the mean. It was found (see Figures 2–4) that students with high intrinsic values showed stronger positive effects of: (a) control beliefs on subjective exercise experience (high: β = 0.115 vs. low: β = 0.036, ns), (b) control beliefs on physical exercise (high: β = 0.225 vs. low: β = 0.097), and (c) subjective exercise experience on physical exercise (high: β = 0.170 vs. low: β = 0.036, ns). These results collectively indicate that intrinsic value amplifies the motivational pathway from cognitive factors to exercise behavior.

**Table 4:** Moderated mediation regression.

Dimension	CB			SEE			PE		
	β	SE	*t*	β	SE	*t*	β	SE	*t*
Gender	−0.038	0.019	−2.015^*^	−0.043	0.021	−2.054^*^	−0.134	0.023	−5.841^***^
Grade	0.158	0.018	8.633^***^	−0.016	0.021	−0.765	−0.011	0.023	−0.469
RR	−0.052	0.019	−2.753^**^	0.046	0.021	2.208^*^	−0.013	0.023	−0.563
IH	0.006	0.019	0.312	0.028	0.021	1.332	0.081	0.023	3.526^***^
PF	0.042	0.020	2.119^*^	0.053	0.022	2.457^*^	0.095	0.024	3.981^***^
PS	0.248	0.022	11.420^***^	0.098	0.025	3.960^***^	0.029	0.028	1.038
CB				0.076	0.028	2.670^**^	0.161	0.031	5.137^***^
SEE							0.103	0.029	3.559^***^
IV	0.509	0.023	22.367^***^	0.501	0.029	17.417^***^	0.222	0.035	6.365^***^
PS × IV	0.011	0.015	0.753	0.024	0.020	1.200	−0.001	0.023	−0.025
CB × IV				0.040	0.020	1.962^*^	0.064	0.025	2.581^**^
SEE×IV							0.067	0.024	2.801^**^
*R* ^2^	0.502			0.404			0.278		
*F*	189.023^***^			101.603^***^			48.044^***^		

*p < 0.05, **p < 0.01, ***p < 0.001.

## Discussion

### The influence of peer support on physical exercise

It was found that after controlling for control beliefs and subjective exercise experience variables, peer support remained directly and positively associated with college students’ physical exercise. This aligns with existing research findings (Liu, 2024; Zhang et al., 2022). For example, Liu (2024) found that after controlling for self-efficacy, peer support still significantly enhanced college students’ participation in physical exercise. Although control beliefs and subjective exercise experiences are important psychological factors influencing exercise behavior, the present study confirms that the role of peer support is not achieved exclusively through the mediating path of psychological cognitions or affective experiences, but rather has a direct facilitative effect on exercise behavior. Previous studies have also found that support from peers can directly enhance an individual’s exercise engagement without relying exclusively on the individual’s intrinsic motivation or cognitive appraisal (Cohen and Wills, 1985). College students’ lifestyles and daily behaviors are closely related to those of their peers (Liu, 2024). When college students’ peer support behaviors are stronger, they are more able to receive support such as information and advice from friends around them, emotional support (encouragement), behavioral role models (exemplary), and accompaniment and supervision (Wang et al., 2018), and are motivated to obtain a stronger value emotional identity, which stimulates the enthusiasm of participating in physical exercise (Zou et al., 2023).

### Analysis of control beliefs as a mediating factor

Studies have confirmed that control beliefs serve as a mediator in the relationship between peer support and college students’ physical exercise. This aligns with existing research findings (Liu, 2024; Yu et al., 2021). The association between peer support and physical exercise is mediated by control beliefs, such that peer support is linked to higher self-efficacy, which in turn is linked to greater physical exercise. However, no mediating effect of self-efficacy was observed between teacher support and exercise behavior. This suggests that peer interactions play a more significant role in shaping students’ self-efficacy compared to teacher support. Given that college students spend considerable time engaging with peers—both socially and psychologically—to foster interpersonal bonds and gain acceptance, peer support emerges as a critical determinant of their self-efficacy beliefs. When the college student group receives supportive behaviors such as informational advice and encouragement from peers, it can help college students to establish correct exercise beliefs (Zou et al., 2023), so that college students can take the initiative to stimulate the control beliefs, enrich the exercise cognitive system, and promote the internalization of exercise motivation in exercise stressful events, which can then ensure participation in physical exercise (Dong and Mao, 2018). Furthermore, the study revealed that the mediating effect of control beliefs was approximately quadruple that of subjective exercise experience, aligning with prior findings by Chen et al. (2017). These results underscore the predominant mediating role of control beliefs in the relationship between peer support and college students’ physical exercise.

### Analysis of subjective exercise experience as a mediating factor

While control beliefs demonstrate significant mediating effects, the contribution of subjective exercise experience remains noteworthy. Empirical evidence confirms its intermediary role in the relationship between peer support and college students’ physical exercise, corroborating existing theoretical perspectives (Xu and Dong, 2020; Tian and Shi, 2022). That is, peer relationships positively predict subjective exercise experience (Xu and Dong, 2020), and subjective exercise experience shows a positive association with students’ leisure-time physical exercise patterns (Tian and Shi, 2022). Research indicates that collegiate individuals receiving athletic companionship, assistance, and encouragement from peers typically report more favorable subjective exercise experience (Cheng and Dong, 2018). These students demonstrate enhanced capacity to evaluate exercise’s benefits for personal development, self-perception refinement, and social adjustment improvement, consequently exhibiting greater propensity for consistent physical exercise engagement (Cheng and Dong, 2018). In contrast, college students who are less likely to perceive peer exercise friendship and autonomy support often feel negative subjective experience and psychological fatigue when engaging in leisure physical exercise (Liu et al., 2011), and thus are difficult to form positive, stable, and appropriate exercise behaviors. Empirical studies suggest that peer support contributes significantly to strengthening undergraduates’ group affiliation and behavioral consistency (Zou et al., 2023). These psychological factors have been shown to positively influence students’ subjective exercise experience during recreational activities, thereby fostering more consistent, health-oriented, and self-congruent physical exercise patterns (Zhang et al., 2023).

### Chain mediation analysis involving control beliefs and subjective exercise

The study established a sequential mediation pathway in which control beliefs and subjective exercise experience collectively mediate the relationship between peer support and college students’ physical exercise. This finding carries important implications, suggesting that peer support is associated with higher levels of exercise-related control beliefs (Zou et al., 2023), which are associated with more positive subjective exercise perceptions (Zhang et al., 2023). These positive perceptions are, in turn, associated with greater physical exercise engagement (Cheng and Dong, 2018). While prior research has well established the chain-mediated relationship between exercise enjoyment and self-efficacy (Chen et al., 2017), with enjoyment preceding self-efficacy, the current study extends this understanding by demonstrating that control beliefs similarly precede subjective exercise experiences in the mediation sequence. Furthermore, these results align with longitudinal evidence showing that interventions enhancing physical activity enjoyment in high school girls subsequently increased their self-efficacy, leading to greater activity levels (Dishman et al., 2005).

### Analysis of intrinsic value as a moderating factor

This study incorporates intrinsic value as a moderating variable into the model, with its theoretical basis mainly derived from Self-Determination Theory and Control-Value Theory. Self-Determination Theory posits that intrinsic motivation (i.e., intrinsic value) is a core internal resource for individuals to persist in a certain activity. It not only directly promotes behavioral persistence but also enhances the psychological connection between an individual’s perception of their own abilities (such as control beliefs) and emotional experiences during the activity (such as subjective exercise experience) (Deci and Ryan, 2000). Control-Value Theory further clarifies that an individual’s emotions and behaviors are not only influenced by control appraisals (such as “Can I do it?”) but also regulated by value appraisals (such as “Is this meaningful to me?”); the interaction of the two jointly predicts emotional responses and behavioral engagement (Pekrun, 2006). Therefore, when an individual holds a high intrinsic value for physical exercise, their control beliefs are more likely to be transformed into positive emotional experiences and further into actual exercise behaviors, with intrinsic value playing the role of a “psychological amplifier” in this process.

Interestingly, intrinsic value failed to moderate peer support’s effects. Theoretically, peer support acts as an external social resource with relatively direct and universal mechanisms, largely independent of individual differences in intrinsic motivation. According to the functional theory of social support (Cohen and Wills, 1985), instrumental and emotional support primarily facilitate behavioral change by providing resources, alleviating stress, or enhancing a sense of belonging, without necessarily requiring filtration through an individual’s intrinsic value system. Thus, peer encouragement, companionship, or modeling can directly strengthen control beliefs or exercise experiences, regardless of whether an individual values the intrinsic aspects of exercise—potentially explaining why intrinsic value did not significantly moderate these pathways. In terms of measurement, the peer support scale used in this study emphasized emotional support and behavioral companionship but inadequately captured “value-congruent support,” which relates to shared meaning, enjoyment, or self-realization in exercise. Including more value-oriented items might reveal a stronger interaction with intrinsic value.

### Implications and limitations

First, intervention strategies designed to promote physical exercise among college students should incorporate peer support elements. Specifically, strategies should include fostering positive peer relationships, encouraging peers to reinforce and model physical exercise (Chen, 2022), establishing peer fitness groups and online communities, exercising with peers, providing mutual encouragement and support, and sharing exercise experiences and achievements (Chen, 2020). These strategies can enhance college students’ control beliefs and subjective exercise experiences when participating in physical exercise, and ultimately increase college students’ physical exercise time. Second, particular emphasis should be placed on enhancing college students’ control beliefs, as they serve as a potent mediator between peer support and physical exercise. Furthermore, control beliefs demonstrate the capacity to influence subjective exercise experiences, thereby indirectly affecting exercise participation. Students should realize that exercise is a gradual process of progress and that they do not have to demand immediate perfection. Celebrate small achievements, i.e., give yourself positive feedback and rewards every time you reach a small goal or make a little progress to enhance self-confidence and sense of accomplishment (Liu, 2024). Regularly record information such as exercise time, intensity, and feelings in order to monitor their progress and adjust their exercise program. Physical education instructors should implement evidence-based pedagogical approaches to simultaneously develop students’ motor competencies and enhance their exercise-related self-beliefs (Shen et al., 2022). This dual-focus methodology promotes sustained physical exercise participation through cultivated confidence in one’s movement capabilities. Again, interventions should also emphasize the subjective experience of physical exercise. This is achieved by maintaining positive thoughts during exercise, focusing on bodily sensations and breathing, and reducing distractions to improve the focus and effectiveness of exercise. Exercise will be utilized as an avenue for emotional release, and negative emotions such as stress and anxiety will be relieved through exercise. Creating a relaxing atmosphere (e.g., comfortable activity space and playing favorite music) enhances the pleasure of exercise (Dishman et al., 2005). Finally, there is also a need to emphasize the development of students’ intrinsic value. Educators and health promoters can enhance students’ intrinsic value by emphasizing the enjoyment, autonomy, and personal significance (e.g., health benefits, skill enhancement) of physical exercise to increase exercise adherence. While enhancing control beliefs (e.g., self-efficacy training), the focus should be on fostering positive exercise experiences (e.g., through fun exercise design), which is especially critical for low intrinsic value groups. College students need to develop their own exercise preferences and should be offered a variety of exercise options to choose from (Shen et al., 2022).

This study has the following shortcomings: (1) This cross-sectional study precludes causal inferences; longitudinal or experimental designs are needed to establish temporal relationships. (2) Peer support—encompassing material, informational, and emotional dimensions—differentially influences collegiate physical exercise; future studies should examine these distinct effects comparatively. (3) The research results have cross-cultural reference value at the theoretical level, but empirical promotion requires caution. Since the samples mainly come from the group of Chinese college students, there may be the influence of culture-specific factors. Convenience sampling may lead to differences in aspects such as academic pressure and availability of campus sports facilities between the sample and the overall group of college students.

## Conclusion

This study elucidates the multifaceted mechanisms through which peer support influences college students’ physical exercise. The findings demonstrate a direct positive association of peer support with exercise participation, along with three significant indirect pathways: (1) the independent mediation of control beliefs (enhancing self-perceived exercise competence), (2) the independent mediation of subjective exercise experience (amplifying affective responses), and (3) their sequential mediation (control beliefs → subjective experience). Importantly, intrinsic value emerged as a critical moderator, intensifying the effects of both control beliefs on subjective experience and their joint influence on exercise behavior. These results extend social cognitive theory by revealing how peer interactions simultaneously shape cognitive appraisals, emotional rewards, and behavioral outcomes in physical activity contexts. Practically, the study suggests that college-based exercise interventions should strategically combine peer support systems with cognitive-affective skill development, while tailoring approaches for students with varying levels of intrinsic motivation. Future research should employ longitudinal designs to verify causality and explore how these mechanisms operate across different exercise modalities and campus environments.

## Data Availability

The raw data supporting the conclusions of this article will be made available by the authors, without undue reservation.

## References

[ref1] Bandura A. (2001). Social cognitive theory: an agentic perspective. Annu. Rev. Psychol. 52, 1–26. doi: 10.1146/annurev.psych.52.1.1, 11148297

[ref2] Chen Z. Y. (2020). The Effect of Peer Support on College Students' Interpersonal Adaptation. Master's thesis. Henan: Henan University.

[ref3] Chen Z. (2022). The mediating and buffering effect of self-control on the relationship between peer support and academic burnout. Eur. J. Hum. Soc. Sci. 6, 71–80. doi: 10.29013/EJHSS-22-6-180

[ref4] Chen S. P. Li S. Z. Yan Z. L. (2006). Research on mechanism of exercise persistence based on sport commitment theory. Sports Sci. 26, 48–55. doi: 10.3969/j.issn.1000-677X.2006.12.009

[ref5] Chen H. Sun H. Dai J. (2017). Peer support and adolescents' physical activity: the mediating roles of self-efficacy and enjoyment. J. Pediatr. Psychol. 42, 569–577. doi: 10.1093/jpepsy/jsw103, 28158660

[ref6] Cheng Y. F. Dong B. L. (2018). Influence of exercise atmosphere and subjective experience on leisure physical exercise of undergraduates. J. Tianjin Univ. Sport. 33, 177–184. doi: 10.13297/j.cnki.issn1005-0000.2018.02.012

[ref7] Cohen S. Wills T. A. (1985). Stress, social support, and the buffering hypothesis. Psychol. Bull. 98, 310–357. doi: 10.1037/0033-2909.98.2.310, 3901065

[ref8] Deci E. L. Ryan R. M. (2000). The “what” and “why” of goal pursuits: human needs and the self-determination of behavior. Psychol. Inq. 11, 227–268. doi: 10.1207/S15327965PLI1104_01

[ref9] Dishman R. K. Motl R. W. Saunders R. Felton G. Ward D. S. Dowda M. . (2005). Enjoyment mediates effects of a school-based physical-activity intervention. Med. Sci. Sports Exerc. 37, 478–487. doi: 10.1249/01.MSS.0000155391.62733.A7, 15741848

[ref10] Dong B. L. Mao L. J. (2018). Parental autonomy support and adolescents' exercise adherence: multiple mediation of control beliefs and exercise involvement. J. Tianjin Univ. Sport. 33, 44–51. doi: 10.13297/j.cnki.issn1005-0000.2018.01.008

[ref11] Eccles J. S. Wigfield A. (2002). Motivational beliefs, values, and goals. Annu. Rev. Psychol. 53, 109–132. doi: 10.1146/annurev.psych.53.100901.135153, 11752481

[ref12] Efrat M. W. (2009). The relationship between peer and / or friends' influence and physical activity among elementary school children: a review. Calif. J. Health Promot. 7, 48–61. doi: 10.32398/cjhp.v7iSI.2000

[ref13] Fernández-Zabala A. Ramos-Díaz E. Rodríguez-Fernández A. Núñez J. L. (2020). Sociometric popularity, perceived peer support, and self-concept in adolescence. Front. Psychol. 11:594007. doi: 10.3389/fpsyg.2020.594007, 33324296 PMC7726329

[ref14] Garn A. C. Simonton K. L. (2022). Motivation beliefs, emotions, leisure time physical activity, and sedentary behavior in university students: a full longitudinal model of mediation. Psychol. Sport Exerc. 58:102077. doi: 10.1016/j.psychsport.2021.102077

[ref15] Gruber K. J. (2008). Social support for exercise and dietary habits among college students. Adolescence 43, 557–575. 19086670

[ref16] Hayes A. F. (2013). Introduction to mediation, moderation, and conditional process analysis: A regression-based approach. New York, NY: Guilford Press.

[ref17] He L. Li Y. Chen Z. (2022). The effect of subjective exercise experience on exercise behavior and amount of exercise in children and adolescents: the mediating effect of exercise commitment. Int. J. Environ. Res. Public Health 19:10829. doi: 10.3390/ijerph191710829, 36078545 PMC9518043

[ref18] Jang H. Kim E. J. Reeve J. (2016). Why students become more engaged or more disengaged during the semester: a self-determination theory dual-process model. Learn. Instr. 43, 27–38. doi: 10.1016/j.learninstruc.2016.01.002

[ref19] Kaushal N. Bérubé B. Hagger M. S. Bherer L. (2021). Investigating the role of self-control beliefs in predicting exercise behaviour: a longitudinal study. Br. J. Health Psychol. 26, 1155–1175. doi: 10.1111/bjhp.12525, 33870633

[ref20] Kwan B. M. Bryan A. (2010). In-task and post-task affective response to exercise: translating exercise intentions into behaviour. Br. J. Health Psychol. 15, 115–131. doi: 10.1348/135910709X433267, 19397847 PMC12240157

[ref21] Liang D. Q. (1994). The stress level of college students and its relationship with physical exercise. Chin. Ment. Health J. 8, 5–6.

[ref22] Liu S. (2024). The Effect of Peer Support on Physical Exercise Behavior of College Students: The Mediating Effect of Self-Efficacy. Master's thesis. Wuhan: Wuhan Sports University.

[ref23] Liu F. Li N. (2023). The influence of sport motivation on college students' subjective exercise experience: a mediation model with moderation. Front. Psychol. 14:1219484. doi: 10.3389/fpsyg.2023.1219484, 37720647 PMC10501446

[ref24] Liu W. N. Zhou C. L. Sun J. (2011). Effect of outdoor sport motivation on sport adherence in adolescents -the mediating mechanism of sport atmosphere. China Sport Sci. 31, 41–47. doi: 10.16469/j.css.2011.10.006

[ref25] Marsh H. W. Martin A. J. Jackson S. (2010). Introducing a short version of the physical self description questionnaire: new strategies, short-form evaluative criteria, and applications of factor analyses. J. Sport Exerc. Psychol. 32, 438–482. doi: 10.1123/jsep.32.4.438, 20733208

[ref26] Menescardi C. Estevan I. (2021). Parental and peer support matters: a broad umbrella of the role of perceived social support in the association between children's perceived motor competence and physical activity. Int. J. Environ. Res. Public Health 18:6646. doi: 10.3390/ijerph18126646, 34205557 PMC8296426

[ref27] Pekrun R. (2006). The control-value theory of achievement emotions: assumptions, corollaries, and implications for educational research and practice. Educ. Psychol. Rev. 18, 315–341. doi: 10.1007/s10648-006-9029-9

[ref28] Pekrun R. Perry R. P. (2014). “Control-value theory of achievement emotions” in International handbook of emotions in education. eds. Pekrun R. Linnenbrink-Garcia L. (New York, NY: Routledge), 120–141.

[ref29] Schiefele U. Krapp A. Winteler A. (1988). Conceptualization and measurement of interest. Paper presented at the annual meeting of the American Educational Research Association. New Orleans, LA. Available online at: https://files.eric.ed.gov/fulltext/ED292885.pdf (Accessed June 15, 2025).

[ref30] Shen L. Chen Y. Sun H. C. (2022). A study on the effect of peer support on physical activity of high school students from border regions-the mediating roles of self-efficacy and enjoyment. Sports Sci. Res. 26, 78–86. doi: 10.19715/j.tiyukexueyanjiu.2022.06.012

[ref31] Siedlecki K. L. Salthouse T. A. Oishi S. Jeswani S. (2014). The relationship between social support and subjective well-being across age. Soc. Indic. Res. 117, 561–576. doi: 10.1007/s11205-013-0361-4, 25045200 PMC4102493

[ref32] Simonton K. L. Garn A. C. (2020). Control–value theory of achievement emotions: a closer look at student value appraisals and enjoyment. Learn. Individ. Differ. 81:101910. doi: 10.1016/j.lindif.2020.101910

[ref33] Simonton K. L. Washburn N. Prior L. F. Shiver V. N. Fullerton S. Gaudreault K. L. (2022). A retrospective study on students' perceived experiences in physical education: exploring beliefs, emotions, and physical activity outcomes. J. Teach. Phys. Educ. 42, 274–282. doi: 10.1123/jtpe.2021-0288

[ref34] Staples C. Palermo M. Rancourt D. (2022). Intrinsic and extrinsic motivations as moderators of the association between exercise frequency and exercise behavior. Eat. Weight Disord. 27, 2801–2809. doi: 10.1007/s40519-022-01430-6, 35776380

[ref35] Tian Y. Shi Z. (2022). The relationship between social support and exercise adherence among Chinese college students during the COVID-19 pandemic: the mediating effects of subjective exercise experience and commitment. Int. J. Environ. Res. Public Health 19:11827. doi: 10.3390/ijerph191811827, 36142099 PMC9517627

[ref36] Tilga H. Kalajas-Tilga H. Hein V. Raudsepp L. Koka A. (2018). The effect of peers' autonomy-supportive behaviour on adolescents' psychological need satisfaction, intrinsic motivation and objectively measured physical activity. Acta Kinesiol. Univ. Tartu. 24, 27–41. doi: 10.12697/akut.2018.24.02

[ref37] Wang K. Pan Y. Q. Lv M. H. (2023). Analysis on moderating effect of extroversion between social support and physical activity among college students in a university in Beijing. Med. Soc. 36, 78–82. doi: 10.13723/j.yxysh.2023.03.014

[ref38] Wang F. B. H. Wang Y. C. Tan M. H. (2018). The influence of partner support behavior on adolescents' physical activity. Chin. Sport Sci. Technol. 54, 18–24. doi: 10.16470/j.csst.201805003

[ref39] Wen Z. L. Ye B. J. (2014). Analyses of mediating effects: the development of methods and models. Adv. Psychol. Sci. 22, 731–745. doi: 10.3724/SP.J.1042.2014.00731

[ref40] Xu L. L. Dong B. L. (2020). Relationship between peer relationship, subjective exercise experience, and adolescents' leisure physical exercise habits: a cross-lagged analysis. J. Tianjin Univ. Sport. 35, 697–702. doi: 10.13297/j.cnki.issn1005-0000.2020.06.013

[ref41] Yang S. J. (2016). Relationship between social support, self-efficiency and satisfaction of youth physical activity. J. Wuhan Inst. Phys. Educ. 50, 90–94. doi: 10.15930/j.cnki.wtxb.2016.02.015

[ref42] Yang H. M. (2017). The study on the actuality and influencing factors of physical activity among college students-taking several universities in Changsha as examples. Master's thesis. Changsha: Hunan Normal University.

[ref43] Yu K. H. Lu Y. J. Wu Y. Z. (2021). An analysis of the structural equation model with the factors affecting exercise behaviors for university students. J. Phys. Educ. 28, 103–110. doi: 10.16237/j.cnki.cn44-1404/g8.2021.02.017

[ref44] Zhang Y. Hasibagen Zhang C. (2022). The influence of social support on the physical exercise behavior of college students: the mediating role of self-efficacy. Front. Psychol. 13:1037518. doi: 10.3389/fpsyg.2022.1037518 36532973 PMC9756807

[ref45] Zhang T. Li H. Y. (2023). An analysis of structural equation model on promotion for physical exercise behavior among teenagers-based on the theory of achievement emotion control value. J. Phys. Educ. 30, 67–75. doi: 10.16237/j.cnki.cn44-1404/g8.20230721.002

[ref46] Zhang T. Li B. He X. Jia P. Ye Z. (2025). The effect of exercise atmosphere on college students' physical exercise-a moderated chain mediation model. Behav. Sci. 15:507. doi: 10.3390/bs15040507, 40282128 PMC12024406

[ref47] Zhang T. Zhao J. Yu L. (2023). The effect of fitness apps usage intensity on exercise adherence among Chinese college students: testing a moderated mediation model. Psychol. Res. Behav. Manag. 16, 1485–1494. doi: 10.2147/PRBM.S408276, 37138699 PMC10150761

[ref48] Zou Y. Liu S. Guo S. Zhao Q. Cai Y. (2023). Peer support and exercise adherence in adolescents: the chain-mediated effects of self-efficacy and self-regulation. Children 10:401. doi: 10.3390/children10020401, 36832530 PMC9955246

